# Dexamethasone Coanalgesic Administration in Steroid Naïve and Steroid Non-Naïve Patients for the Prevention of Pain Flares after Palliative Radiotherapy for Bone Metastases

**DOI:** 10.1155/2022/6153955

**Published:** 2022-11-28

**Authors:** Dimitar G. Tonev, Silvia A. Lalova, Elena P. Petkova-Lungova, Nikolay V. Timenov, Gabriela M. Radeva, Todor G. Kundurzhiev

**Affiliations:** ^1^Medical University of Sofia, Sofia, Bulgaria; ^2^Clinic of Anesthesiology and Intensive Care, University Hospital “Tsaritsa Joanna-ISUL”, Sofia, Bulgaria; ^3^Radiotherapy Clinic, National Oncology Hospital, Sofia, Bulgaria; ^4^Department of Anesthesiology and Intensive Care, National Oncology Hospital, Sofia, Bulgaria; ^5^Medical Oncology Clinic, University Hospital “Tsaritsa Joanna-ISUL”, Sofia, Bulgaria; ^6^Faculty of Public Health, Medical University of Sofia, Sofia, Bulgaria

## Abstract

**Objective:**

Dexamethasone could be an effective prophylactic agent for the prevention of pain flares after palliative radiotherapy (RT) for uncomplicated bone metastases. To date, there are no data on its prophylactic coanalgesic (opioid-sparing) effect after RT in patients with complicated bone metastases compared to uncomplicated ones, which is the aim of our study.

**Methods:**

Twenty-nine American Society of Anaesthesiologists (ASA) III-IV patients, aged ≥18, treated with single-fraction 8 Gy/1 or multi-fraction 20 Gy/5 RT for painful uncomplicated bone metastases (steroid naïve patients, *n* = 14) or complicated ones (steroid non-naïve patients, *n* = 15), were examined retrospectively. All patients received parenteral dexamethasone (4 mg or 8 mg daily, 1 hour before RT, followed by the same dose for the next 4 days) along with their background and breakthrough pain opioid intake (morphine equivalents) during their 5-day in-hospital stay. Pain severity (numeric rating scale) and analgesic consumption were recorded at admission, daily during the hospital stay, and for 10 days following treatment. Binary logistic regression was used to determine predictive factors for pain flare occurrence.

**Results:**

A higher ASA score is the only determinant positively influencing opioid consumption (*P* = 0.018) and pain flare as well (OR = 15.00; 95% CI: 2, 24–100, 48; *P* = 0.005). Lower dose 4 mg dexamethasone was revealed as a moderate analgesic agent in steroid naïve patients with no side effects, whereas in steroid non-naïve patients the predominantly higher dose 8 mg dexamethasone had minimal impact on pain flares prevention at the expense of more pronounced immunosuppression (*P* = 0.039).

**Conclusions:**

Irrespective of the supporting evidence of dexamethasone potential for prevention of RT-induced pain flare, our data failed to reveal its efficacy in the real practice world (a case mix of uncomplicated and complicated bone metastases). Further dose-effect bigger studies are needed, identifying optimal doses of dexamethasone intake and its optimal duration in high-risk patients.

## 1. Introduction

Bone metastases are a frequent occurrence in advanced cancer, especially cancers of the breast, prostate, and lung. Metastatic bone destruction leads to increased morbidity and an impaired quality of life as a result of pain, fractures, and other skeletal-related events. Not all patients with bone metastases are symptomatic, but up to 70% of patients with metastatic bone disease experience severe pain [1]. For patients with advanced cancer and painful bone metastases, radiation therapy (RT) is an effective palliative treatment, with about 62% responding within 3 to 4 weeks after treatment [2].

After RT for painful bone metastases, up to 44% of patients report pain flares [3]. Dexamethasone could be an effective prophylactic agent for the prevention of pain flares after palliative radiotherapy for uncomplicated bone metastases [4]. To date, there are no data on its prophylactic c-analgesic (opioid-sparing) effect after RT in patients with complicated bone metastases compared to uncomplicated ones, which is the aim of our study.

## 2. Materials and Methods

Twenty-nine American Society of Anaesthesiologists (ASA) III-IV patients, aged ≥18, treated with single-fraction 8 Gy/1 or multi-fraction 20 Gy/5 RT for painful uncomplicated bone metastases (steroid naive patients, *n* = 14) or complicated ones (steroid non-naive patients, *n* = 15), were examined retrospectively. The inclusion criteria included patients aged ≥18 who had pathologically proven primary solid malignancy with uncomplicated or complicated bone metastases, with indications for single or multiple-fraction radiotherapy and corticosteroid coanalgesic administration, with pain intensity on a numeric rating scale (NRS) ≥2 and able to give a verbal pain score along with data concerning their analgesic consumption on admission, during the hospital stay and follow-up. The exclusion criteria included patients who had not completed the planned course of radiotherapy, with contraindications to steroids such as uncontrolled hypertension, diabetes, infection, or an active peptic ulcer, with painless bone metastases, as well as with incomplete medical records regarding the assessment of pain and analgesic consumption. All patients received parenteral dexamethasone (4 mg or 8 mg daily, 1 hour before RT, followed by the same dose for the next 4 days), along with their background and breakthrough pain opioid (morphine equivalents) and/or nonopioid analgesics (in case of NSAIDs coupled with PPI), which have already been prescribed before the admission and continued until the end of the follow-up. Data regarding demographics, the eastern cooperative oncology group (ECOG) [5] and ASA scores [6], the comorbidities, the primary cancer site, the index site of radiated bone lesions, the CBC, and side effects were collected as well. Pain severity (numeric rating scale (NRS) [7]) and analgesic consumption data were recorded by the medical staff at admission, daily during the hospital stay, and for 10 days following RT.

### 2.1. Definitions

Uncomplicated bone metastases were defined as metastatic tumour masses without massive infiltration towards soft tissue, characterized by a low risk of imminent pathological fracture and no evidence of spinal cord compression or cauda equina compression, and which were not previously irradiated [8]. Complicated bone metastases were defined as bone metastases associated with a soft tissue mass or extraosseous component, neuropathic pain, the need for postsurgical RT, or an impending or high fracture risk in weight-bearing bones [9].

Pain flares were defined as a worsening of basal pain, recorded as a 2-point increase in the NRS of 0–10 (0 meaning no pain, 10 meaning the worst possible pain), without a reduction in the analgesic intake or an increase of 25 percent of the analgesic intake without an improvement of the worst pain score [10]. Pain score and analgesic intake must have returned to baseline during the 10 days after RT to differentiate a pain flare from pain progression [3]. For patients who were not using opioid analgesics in the 24 hours prior to receiving RT, consumption of any opioid analgesic without a reduction in the worst pain score relative to the worst pain score prior to treatment was also considered a pain flare as well [9].

### 2.2. Endpoints

Primary endpoints were the assessment of coanalgesic opioid-sparing effect, efficacy, and safety of implemented dexamethasone prophylaxis in steroid naïve and steroid non-naïve patients. The secondary end point was to determine which factors among demographic, clinical, and treatment variables were associated with the occurrence of pain flares.

### 2.3. Ethical Considerations

The study protocol for this single-center retrospective observational study was reviewed and approved by the local ethics committee. Owing to the retrospective nature of this study and the anonymization of data, the need for informed consent was waived. Another consideration for the waiver was that all the studied patients had already given signed informed consent to begin in-hospital treatment. The study was performed according to the Declaration of Helsinki principles and the EU General Data Protection Regulation.

### 2.4. Statistical Analysis

Data were analyzed using SPSS v.20 (IBM SPSS, Armonk, NY). Categorical data were compared using the *χ*^2^ test or Fisher's exact test (as appropriate). Continuous variables were compared with independent samples *T*-test in normal distribution or Mann–Whitney *U* test for distribution of the different from the normal (between-group comparisons) and with paired samples *T*-test or Wilcoxon Signed Ranks *Z*-test, respectively (within-group comparisons). Binary logistic regression was used to determine predictive factors for pain flare occurrence. All statistical analyzes were performed at *α* = 0.05.

## 3. Results

**Figure 1:**
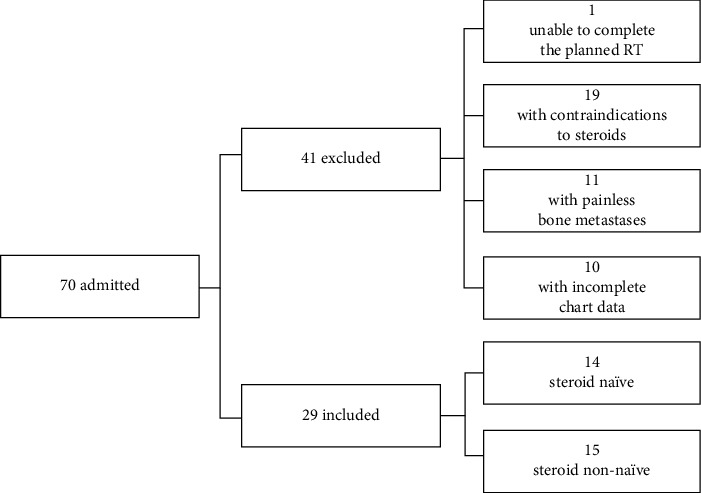
Flow diagram of the population assessed for eligibility, excluded patients, and included patients evaluated for pain flares and responses to dexamethasone prophylaxis.

Within 1 year, a total of 70 patients were admitted, 41 were excluded, and 29 charts were abstracted (Figure 1).

There were no between-group differences in terms of demographics, ECOG, ASA, comorbidities, malignancy, implemented RT, or analgesic consumption (Table 1). There was a tendency for more primary breast cancer in the corticosteroid naïve group and more primary cancers from other sites in the corticosteroid non-naïve group (*P* = 0.059).

As can be seen from Table 2 there were no between-group differences in terms of prevention of radiation-induced pain flare and nausea and vomiting, irrespective of significantly higher doses of dexamethasone in steroid non-naïve group compared to steroid naïve group. In total, 10 patients had pain flares (34%), of them 4 in steroid naïve group (28%) and 6 in steroid non-naïve group (40%).

All dexamethasone side effects were in the steroid non-naïve group, with significantly more immediate adverse effects associated with hyperglycemia and immunosuppression (Table 2). The immunosuppressive effect was more pronounced in terms of WBC elevation (G/l) in patients receiving 8 mg of dexamethasone compared to 4 mg of dexamethasone (30.72 ± 37.66 vs. 8.19 ± 5.20; *P* = 0.039).

**Figure 2:**
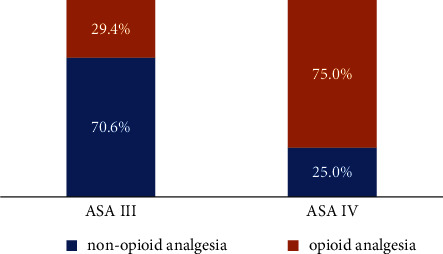
Implemented analgesia in ASA III (*n* = 17) and ASA IV (*n* = 12) patients (%) (*P* < 0.05).

**Figure 3:**
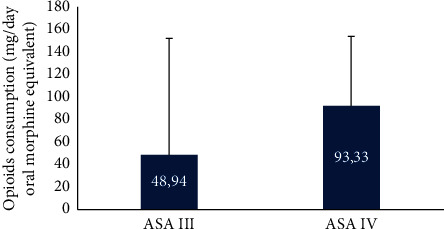
Opioid analgesics consumption in ASA III and ASA IV patients (mg/day OME) (*P* < 0.05).

A higher ASA score is the only determinant positively influencing opioid consumption (Figures 2 and 3) and pain flare as well (binary logistic regression: OR = 15.00; 95% CI: 2, 24–100, 48; *P* = 0.005).

## 4. Discussion

Painful bone cancer metastatic lesions are very challenging to manage due to the etiology of the pain generator involved. The inflammatory process and the constant remodeling activity are the main pain generators at the bony site. However, depending upon the growth of the tumor bed, additional pain generation may occur, such as neuropathic pain secondary to spinal cord compression or nerve root compression [12]. Opioids are currently the mainstay of metastatic bone cancer treatment. In addition to opioids and non-opioid adjuvants, clinicians utilize bisphosphonates, RT, and human monoclonal antibodies for alleviating pain [12], all in line with our local practice as well. Bisphosphonates as potent inhibitors of bone resorption may cause hypocalcemia, which requires supplementation in order to increase bone mineral density, which in turn could decrease the risk of pathological fracture, spinal cord compression, and the associated pain [13]. We found no between-group differences in the received bisphosphonates or in the patients' baseline calcium levels (such as hypocalcemia requiring dietary intake of calcium, vitamin D, or silicon dioxide) that could interfere with our pain assessment and management.

RT is an effective and standard modality for the treatment of painful, complicated, and uncomplicated bone metastases [14]. It is thought to act by reducing local inflammation and causing tumor shrinkage. Anti-inflammatory adjuvants such as corticosteroids (with the corticosteroid of choice, dexamethasone, in advanced uncontrolled bone pain) may also control pain by reducing peritumoral inflammation and edema and mitigating tumor compression and infiltration onto the surrounding tissues and radicular nerves as well, but they do not affect the tumor mass itself. In our study of both uncomplicated and complicated bone metastases, we used conventional external beam RT (EBRT) along with a prophylactic dose of dexamethasone (such as 4 to 8 mg daily for pain flares), not a therapeutic one (such as 16 to 32 mg daily for spinal cord compression).

Multiple studies have demonstrated a single 8 Gy fraction to be as efficacious as a multiple-fraction course for painful bone metastases [15]. Use of single-fraction treatment has been found to be more common in Europe, Canada, and Australia, but it remains the minority [2]. It is even more rarely used, according to studies in the United States [2] and in the present study as well (Table 1). However, in the context of the COVID-19 pandemic, a single 8 Gy fraction should become the default treatment given to painful bone metastases as it minimizes environmental exposure, saves time and is a cost-effective option [16]. Although single-fraction RT is a well-known independent predictor of pain flare (OR = 2.48; 95% CI: 1.23–4.98; *P* = 0.011) [17], it had an equal minimal impact on both groups in our study (Table 1).

The observed pain flare rate in our corticosteroid naïve patients (28%) is in line with that for uncomplicated bone metastases reported by others (24% after EBRT) [18]. However, the pain flare rate in corticosteroid non-naïve patients (40%) is closer to that reported without dexamethasone (44%) [3]; than that with 4 or 8 mg dexamethasone prophylaxis [19]. The lower dose of 4 mg of dexamethasone could be effective as a prophylactic agent in steroid naïve patients with no side effects, whereas in steroid non-naïve patients the observed pain flares suggest that a higher therapeutic dose of dexamethasone could be more relevant than the prophylactic one, especially in high-risk patients. In steroid non-naïve patients were observed only immediate corticosteroid side effects, namely hyperglycemia in one patient and pronounced WBC count elevation in the other three (Table 2).

Our higher dose of 8 mg Dexamethasone prophylaxis was implemented at the expense of more pronounced immunosuppression in terms of WBC count elevation (*P* = 0.039), which to some extent might reflect the more pronounced systemic inflammatory response to tumor activity in complicated bone metastases as well [20]. Given the recent emergence of immunotherapies, it is important to consider that the use of corticosteroids could negatively impact some new immune-related treatments, such as immune checkpoint inhibitors or adoptive cell therapies [10]. Our findings suggest that 4 mg Dexamethasone prophylaxes could be more relevant alternative in corticosteroid naïve patients receiving immunotherapies. Further research should focus not only on the corticosteroid usage during the time of implemented RT but also on the impact of higher dose/single shot (8 Gy/1 RT coverage) versus lower dose/multiple shots (20 Gy/5 RT coverage) dexamethasone prophylaxis as well as on the dose/time optimization during the whole 10-day vulnerable period (in terms of pain flares) after RT.

Data from palliative care settings failed to reveal the opioid-sparing effect of nonopioid analgesics implemented as adjuvants for pain relief (including corticosteroids) on opioid analgesics. The authors suggested further research to compare cancer patients admitted to the in-patient palliative care unit, who are already on adjuvants with those who would start their adjuvants in the hospital [21]. Our study gave the above-proposed comparison between steroid naïve and steroid non-naïve in-hospital patients and confirmed the lack of an opioid-sparing effect in particular case of prophylactic coanalgesic administration of dexamethasone for the prevention of pain flares after palliative RT for symptomatic bone metastases (Table 1). Thus, our findings confirm the latest RCT unconvincing evidence of dexamethasone reduction of the incidence of pain flares in the field [22].

Our patients were treated with nonopioid analgesics, as NSAIDs and paracetamol, which had already been prescribed before the admission and were continued until the end of the follow-up. This could probably mask the dexamethasone opioid-sparing effect, but our nonopioid and opioid consumptions were evenly distributed between the groups (Table 1), and we therefore find it unlikely that this affected the study results.

In all-patients comparison, however, the implemented non-opioid analgesics (including simple analgesics, NSAIDs, and adjuvants) do reveal their opioid-sparing effect in ASA III patients compared to ASA IV patients (Figure 2). The latter finding is probably not a coincidence given the fact that the ASA classification was rated as a strong prognostic factor predicting survival in patients with spinal bone metastases [23], and the use of non-opioid analgesics was an independent prognostic factor in the same patients as well [24].

On the other hand, opioid use itself is associated with an increased hazards of death (HR = 1.59 (95% CI: 1.38–1.84), *P* < 0.001) in patients with advanced cancer [19], almost as much as the severity of pain (HR 1.79 CI 1.43–2.24, *P* < 0.0001) in patients with painful bone metastases [25]. In our study, a higher ASA score was the only determinant influencing positively opioid consumption (Figure 3) and pain flare as well (binary logistic regression: OR = 15.00; 95% CI: 2, 24–100, 48; *P* = 0.005). The resulting wide CI here to some extent are due to the small sample size, which with its retrospective design can be considered as limitations of our study. Another limitation is the implemented EBRT only in patients with painful bone metastases which does not allow a generalization of our results in other modalities of RT such as SBRT.

A recent debate on the use of dexamethasone to prevent pain flares concluded that consensus for routine use has not been achieved. Thus, the choice to use dexamethasone prophylactically should be between radiation oncologists and patients. Factors including symptom burden, comorbidities, performance status, and quality of life as well as the radiation dose and fractionation should be considered on an individual level [22]. We confirm the individual approach, adding new data on the emerging role of the ASA, especially in high-risk patients. The ASA could help in the context of an internationally adopted multidisciplinary algorithm for the management of painful bone metastases [26], in which the surgical perspective is an integral part of that of the medical oncology specialists. It makes the prognosis for survival and choice of palliative treatment more clearly understandable for both the surgeon and other interventional pain medicine specialists involved, e.g., anaesthesiologists, interventional radiologist, etc.

## 5. Conclusions

Irrespective of the supporting evidence in a RCT of dexamethasone potential for prevention of RT-induced pain flares, our data failed to reveal its efficacy in the real practice world (a case mix of uncomplicated and complicated bone metastases). Further dose-effect bigger studies are needed, to identify optimal doses of dexamethasone intake and its optimal duration in high-risk patients.

## Figures and Tables

**Table 1:** Participant demographic, clinical, and treatment data (mean ± SD, unless otherwise specified).

	Steroid naïve (*n* = 14)	Steroid non-naïve (*n* = 15)	*P*
Age (years)	61, 29 ± 14, 63	57, 67 ± 9, 00	0.315
Gender (male/female)	5/9	7/8	0.550
ECOG (0-1/2-3)	5/9	6/9	0.827
ASA (1/2/3/4)	0/0/8/6	0/0/9/6	0.876
Primary cancer site (breast/prostate/lung/other)	9/0/3/2	3/1/4/7	0.059
Index site of radiated bone lesion(s) (cervico-thoracic/lumbo-sacral/other)	7/4/3	3/8/4	0.234
Radiation dose fractionations (8 Gy/1 fr./20 Gy/5 fr.)	2/12	2/13	1.000
Index pain analgesia (nonopioid analgesia/opioid analgesia)	7/7	8/7	1.000
Oral morphine equivalent (OME) (mg/day)	40, 71 ± 67, 17	92, 13 ± 109, 45	0.176
WHO-analgesic ladder (nonopioids/weak opioids/strong opioids)	9/1/4	6/3/6	0.401
Analgesic quantification algorithm [11] (none/weak/strong ≤75 mg/day/strong >75–150 mg/day/strong >150–300 mg/day)	9/1/4/0/0	6/3/3/2/1	0.361
Received bisphosphonates (yes/no)	4/10	2/13	0.390

**Table 2:** Dosage, efficacy, and safety of implemented dexamethasone prophylaxis.

	Steroid naïve (*n* = 14)	Steroid non-naïve (*n* = 15)	*P*
Dexamethasone parenteral dosage (4 mg/8 mg)	13/1	9/6	0.041
Prevention of radiation-induced pain flares (yes/no)	10/4	9/6	0.503
Prevention of radiation-induced nausea and vomiting (yes/no)	8/6	12/3	0.188
Experienced dexamethasone-induced side effects (yes/no)	0/14	4/14	0.039

## Data Availability

The data used to support the findings of this study are available from the corresponding author upon request.
